# Understand how machine learning impact lung cancer research from 2010 to 2021: A bibliometric analysis

**DOI:** 10.1515/med-2023-0874

**Published:** 2024-02-09

**Authors:** Zijian Chen, Yangqi Liu, Zeying Lin, Weizhe Huang

**Affiliations:** Department of Cardiothoracic Surgery, The Second Affiliated Hospital of Shantou University Medical College, Shantou, China

**Keywords:** lung cancer, machine learning, bibliometric analysis, global trend, collaboration, burstiness

## Abstract

Advances in lung cancer research applying machine learning (ML) technology have generated many relevant literature. However, there is absence of bibliometric analysis review that aids a comprehensive understanding of this field and its progress. Present article for the first time performed a bibliometric analysis to clarify research status and focus from 2010 to 2021. In the analysis, a total of 2,312 relevant literature were searched and retrieved from the Web of Science Core Collection database. We conducted a bibliometric analysis and further visualization. During that time, exponentially growing annual publication and our model have shown a flourishing research prospect. Annual citation reached the peak in 2017. Researchers from United States and China have produced most of the relevant literature and strongest partnership between them. *Medical image analysis* and *Nature* appeared to bring more attention to the public. The computer-aided diagnosis, precision medicine, and survival prediction were the focus of research, reflecting the development trend at that period. ML did make a big difference in lung cancer research in the past decade.

## 1 Introduction

Lung cancer has always been an unassailable malignancy with the highest mortality rate in a long history, but maintain a tiny decrease at a pace of 1.5–2% annually in recent years, which accelerated gradually [1]. After decades of stagnation, we witnessed more positive prospects against lung cancer as significant progresses have been made in medical practice, health policy, and smoking cessation over the last 10 years [2–4]. Survival gains is more obvious in non-small cell lung cancer and benefited from early detection, surgical techniques, and targeted therapies, and the advent of immunotherapy brought some hope [5]. Unfortunately, the 60-month overall survival rate for lung cancer remains low, presenting with 68 and 0–10% for stage IB and IVA–IVB patients [6]. In the context of rapid development, it is urgent to understand development trend and research frontier.

Machine learning (ML) is a branch of artificial intelligence that specializes in using mathematical algorithms, recognizing rules in data, and then making forecasts [7]. It is a milestone that ML integrates with medicine activities in the future of healthcare. Lung cancer research is developing in the direction of integrating multiple data types and large scale. But even with the help of dimension reduction methods, analysis of exponentially growing cancer-related databases to accomplish clinical tasks also requires a lot of time and expertise, all these features pose obstacles to clinical application [8]. ML employs statistical analysis to fit an optimal model for particular task in an unsupervised way [9]. Hence, ML is more flexible and scalable than traditional biometric methods, making it powerful to stratify risk, predict prognosis, diagnosis, and classify [10]. As we all know, ML has been involved in three broad areas of biomedicine: clinical diagnostics, precision treatments, and health monitoring [11]. For example, ML improves diagnostic imaging capabilities in terms that predict patient prognosis, inevitably promoting development of radiology medicine [12]. ML generates more reasonable biomarker assessment, and satisfy the accuracy in histopathologic diagnosis of cancer, which further realize individualized treatment [9,13].

There has been an increasing attention between ML and lung cancer over a decade. In general, ML would probably play a huge role in helping medical staff in daily clinical practice of lung cancer in the future; it is exciting to witness its benefits for the patients and doctors from common use [14]. Despite numerous research to explore the role of ML in lung cancer, there is still inadequate global and comprehensive report reflecting main research hotspots and development directions. In the present review, we used a bibliometric analysis to outline a vision as to how ML can impact lung cancer research from 2010 to 2021, with a hope to contribute for a better understanding of ML’s role and potential in this field.

## 2 Methods

### 2.1 Document retrieval and preprocessing

All the documents were retrieved from Clarivate Analytic’s Web of Science Core Collection database (WoSCC) (https://www.webofscience.com/wos/alldb/basic-search) on October 26, 2022, available editions including Science Citation Index Expanded, Social Sciences Citation Index, Arts & Humanities Citation Index, Emerging Sources Citation Index, Emerging Sources Citation Index, and Index Chemicus. The medical subject headings “ML” and “Lung Neoplasms” were adopted for searching strategy. The detail of the query contained was as follows: #1, (ALL = (ML*)) OR ALL = (deep learning*); #2, ((((ALL = (lung Adenocarcinoma*)) OR ALL = (lung cancer*)) OR ALL = (Lung Neoplasm*)) OR ALL = (Pulmonary Cancer*)) OR ALL = (Pulmonary Neoplasm*); #3, “#1,” and “#2.” Publications date was limited between 2010 and 2021, document types were set to articles or reviews, and language was restricted to English. Totally there were 2,464 documents identified. Bibliometric information was fully exported, including address, source, title, keyword, abstract, publication year, time cited count, language, document type, and reference records.

**Figure 1:**
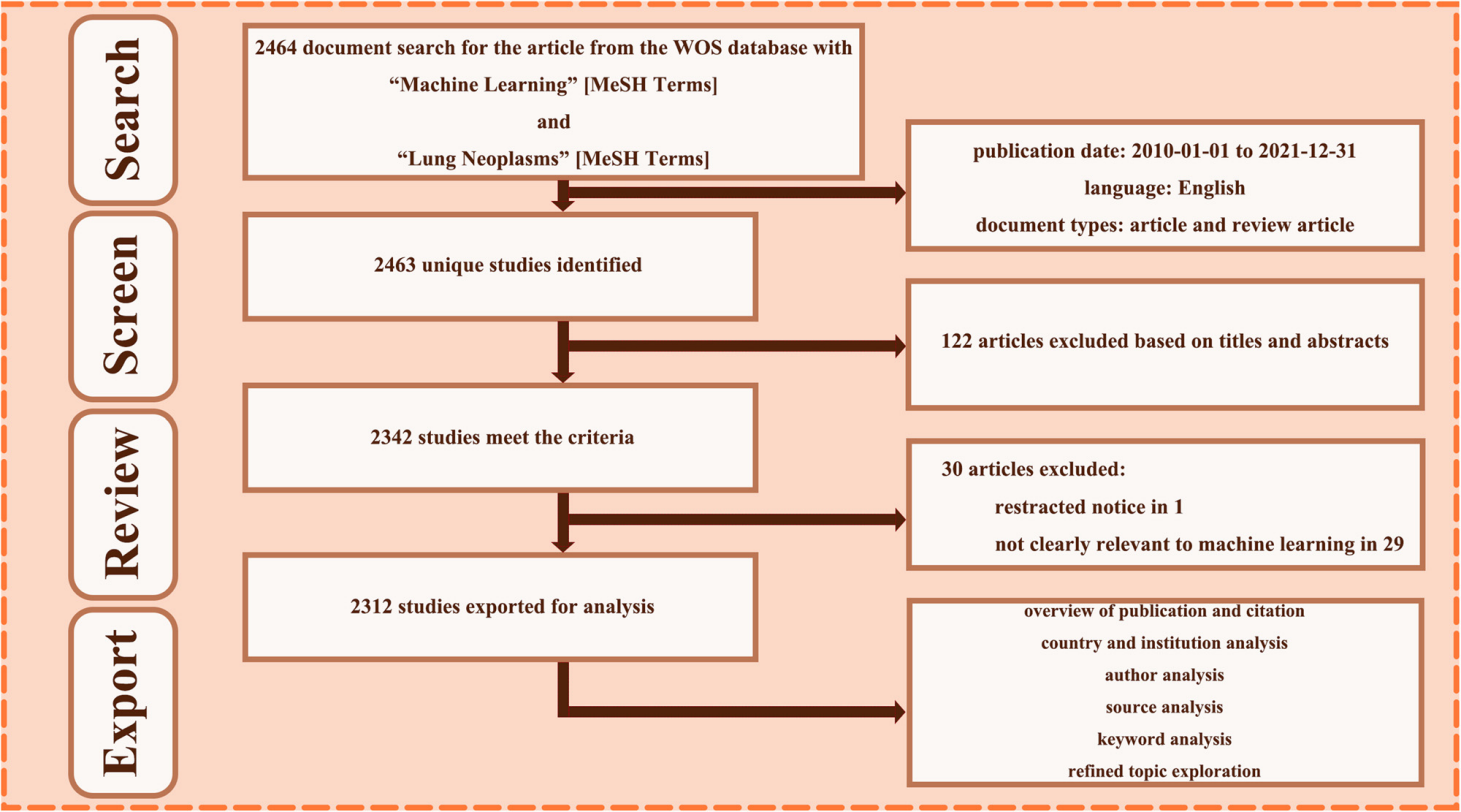
Flowchart of data collection and analysis.

In the preprocessing, CiteSpace V (version 6.1.R2) software was used to organize these voluminous and messy data in an efficient way. After duplicates removing, titles and abstracts of unique 2,463 studies were screened and infiltrated manually. Necessary full-texts review was completed by two authors independently. The inclusion criteria were as follows: (1) a clear correlation with ML, (2) focus on lung cancer research, (3) and not withdrawn for academic controversial. In total, 2,312 studies were exported for downstream bibliometric analysis. The above design framework is shown through a flowchart (Figure 1).

### 2.2 Bibliometric analysis

Biblioshiny is a R tool for scientific mapping analysis run through R package Bibliometrix. Based on raw data from the retrieved WoSCC documents, comprehensive bibliometric analysis was completed, which specifically embraces several modules: overview (annual scientific production, average citations per year), sources (most relevant source), conceptual structure (co-occurrence for keywords plus), and social structure (collaboration network of institution and country). In addition, ethics committee approval was not necessary for this analysis because human or animal subjects is lacking here.

VOSviewer (version 1.6.18) and CiteSpace V (version 6.1.R2) software were applied to realize a series of bibliometric analysis and visualization. Co-authorship analysis for geographical trait like country, institution, overlapping source in cited reference reflects similarities. Keywords co-occurrence analysis reveal the content associations and characteristics of information. Besides, obtaining citation bursts for references and getting the keywords with great change in a specific period. We additionally extracted the highly cited paper (HCP) for further review and classification, and the research networks were analyzed accordingly. Conclusions were then drawn to highlight current situations and predict possible developments in this area.

## 3 Results

### 3.1 Overview of publication and citation

**Figure 2:**
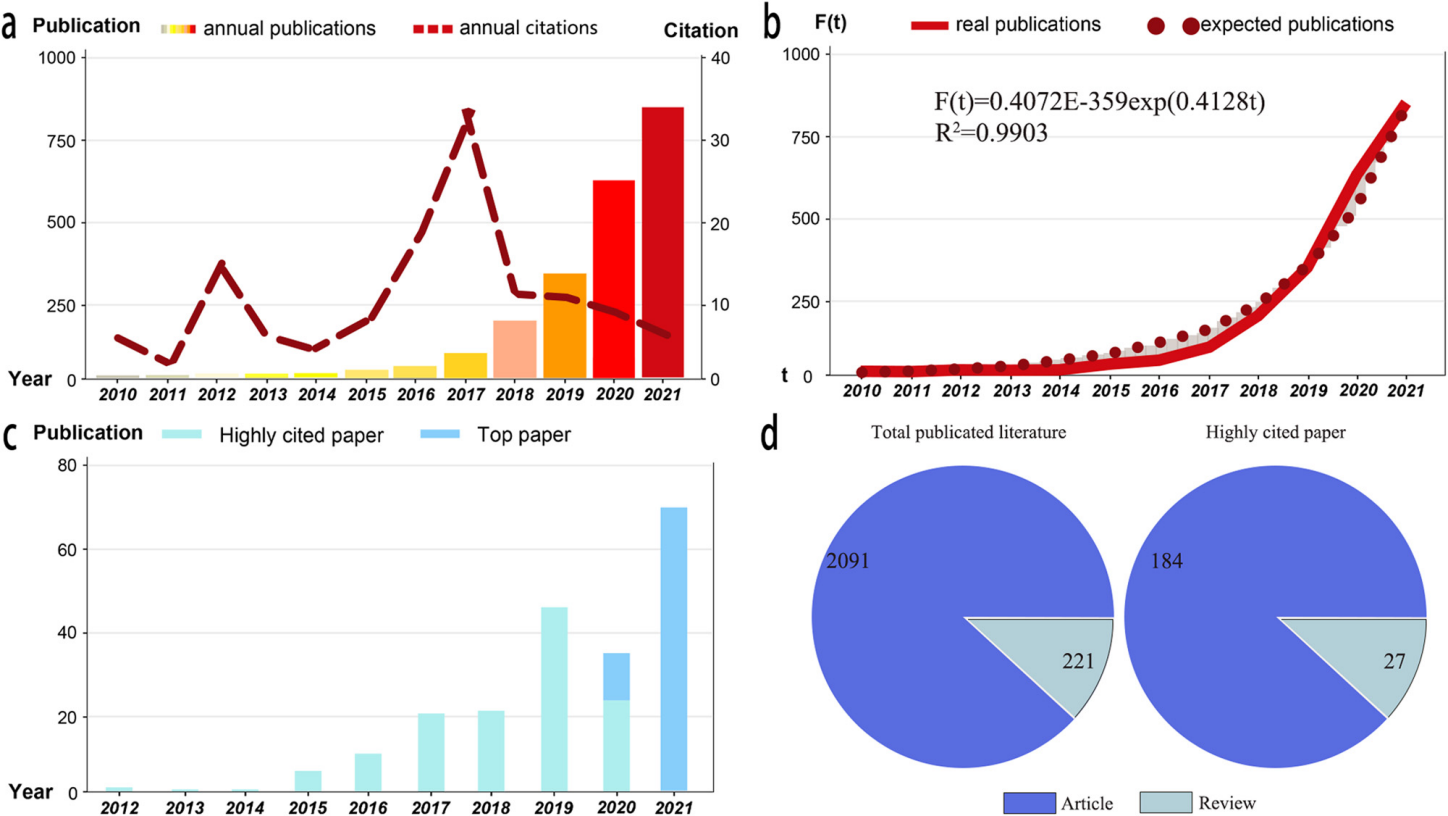
Overview of relevant literature from 2010 to 2021. (a) Annual numbers of publication and citation times of literature. (b) Price’s curve was calculated to predict the trend, with satisfying coefficient of determination of 0.9903, indicating that the annual publication number will continue to grow. (c) Annual numbers of publication of HCP and top paper. (d) Type of total publication literature and HCP.

The annual number of publication and citation from 2010 to 2021 can reflect the research tend. The studies about ML in lung cancer maintained an exponential increase, and reached the maximum in 2021, showing researcher’s rising interest in this topic (Figure 2a). Citation as a periodic index in dynamic state, its fluctuation was relatively smooth and peaked in 2017. To describe the increasing law in some degree, Price’s curve was calculated and the equation is displayed as follows:

\[{[}F(t)=0.4072E\left-359\exp \left(0.4128t)]]\]

High coefficient of determination (*R* ^2^ = 0.9903) indicates that there is an extremely good agreement between the new fitted curve and the real curve (Figure 2b). Through the simulation curve, it can be forecast an increasing annual number of publications soon. Based on the Price’s curve equation, the literature growth at an average annual growth rate of 41.28%, reaching a doubling count in 1.68 years.

Taking the search time as the bound, according to clinical medicine research field threshold of Essential Science Indicators in the version of October 2022, there are 211 HCPs that were ranked in the top 0.1% of the world in terms of citations in the last 10 years (2012–2021) in our study. Rising trend of HCP further proved that ML truly promotes the research progress of lung cancer. Among them, about 83 hot papers are published within the last 2 years, and are referred as “top paper” (Figure 2c). Total publication literature and HCP mainly consist of article, accounting for 90.44 and 97.23%, respectively (Figure 2d).

### 3.2 Country and institution analysis

**Figure 3:**
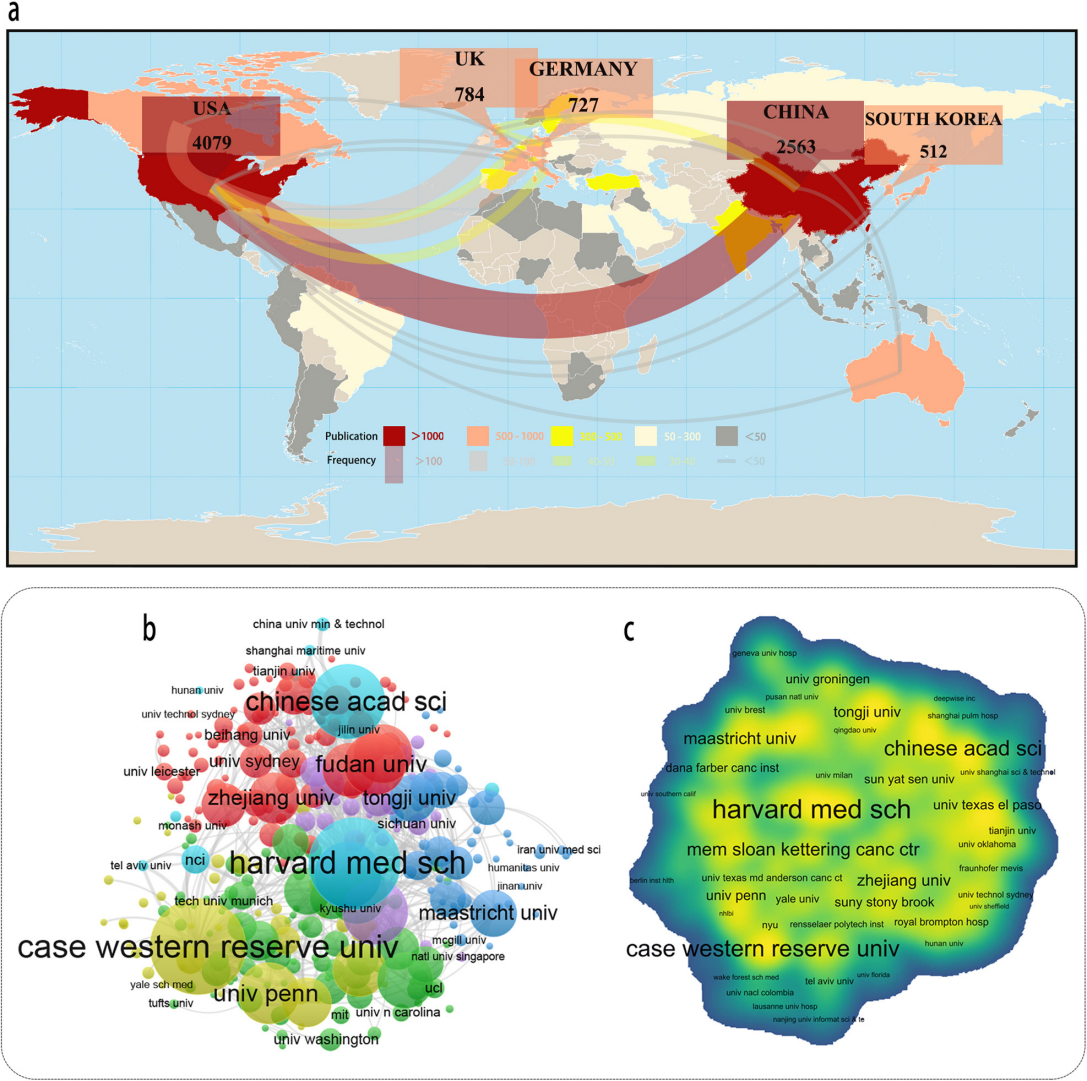
Output and collaboration of relevant literature at country and institution level. (a) Global geographical distribution of publications and collaboration between countries. Co-authorship analysis between institutions: (b) in network map based on publication number and (c) in density map based on their collaboration frequency.

With respect to study output for ML and lung cancer, relevant country’s scientific production and their international cooperation were visualized in a geographic distribution map (Figure 3a). Based on WoSCC data, all together 101 relevant countries/regions and 13,539 relevant research from 2010 to 2021 were identified. At the national level, USA (*n* = 4,079, 30.13% of 13,539) published the most articles, one and a half times more than China (*n* = 2,563, 18.93% of 13,539). In term of HCP, a total of 28 countries/regions were involved in the publication. USA (*n* = 121, 57.35% of 211) contributed the most, China (*n* = 59, 27.96% of 211) ranked behind. In term of citation count, the USA’s literature had highest overall acceptability for their value and originality, and secondarily for China. In term of collaboration, the frequency among them up to 7,966 times, mainly focus on North America (frequency = 1,325, 16.63% of 7,966). Collaboration network revealed that USA plays a crucial role in academic exchange, with positive cooperation with China (frequency = 187, 2.35% of 7,966). From the above publication status, USA relatively held leading status in this topic, followed by developing country China.

Likewise, as many as 4,142 institutions have tried to combine ML and lung cancer (Figure 3b and c). The production of Stanford University (*n* = 117, 0.92% of 12,709) is slightly ahead of other institutions, Harvard Medical School (*n* = 108, 0.85% of 12,709) followed by a small margin. In terms of HCP, there were 363 institutions that participated in the publication, with Harvard Medical School (*n* = 17, 8.06% of 211) leading the list. In terms of collaboration, Harvard Medical School (degree centrality = 1) maintains the most active interaction with other institutions.

### 3.3 Source analysis

**Figure 4:**
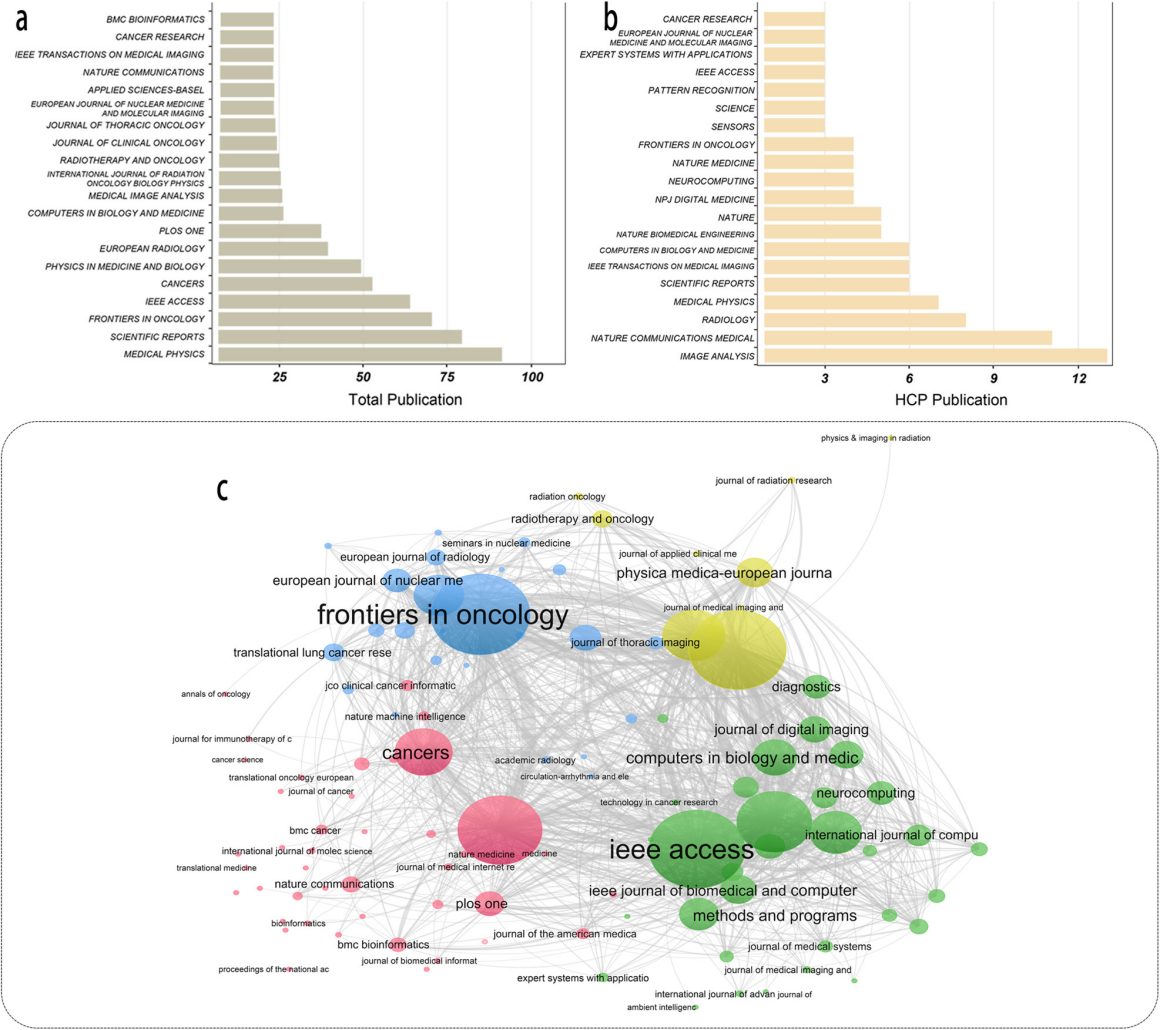
Publication status and co-occurrence analysis of journal source. Publication of (a) total literature and (b) HCPs in journal source. (c) Co-occurrence analysis between journal source in network map.

A total of 2,464 literature were published in 734 journals in this field over the analysis period. *Medical Physics* published the most literature (*n* = 90, 3.7% of 2,464). The second most popular journal was *Scientific Report* (*n* = 79, 3.2% of 2,464). As for HCP section, there are some of the most-influential publication sources like *Medical image analysis* (*n* = 13, 6.2% of 211), *Nature communications* (*n* = 11, 5.2% of 211), *Radiology* (*n* = 8, 3.8% of 211) that support their publication (Figure 4a and b). The co-occurrence analysis map was also achieved from VOSviewer (Figure 4c).

### 3.4 Category analysis

**Figure 5:**
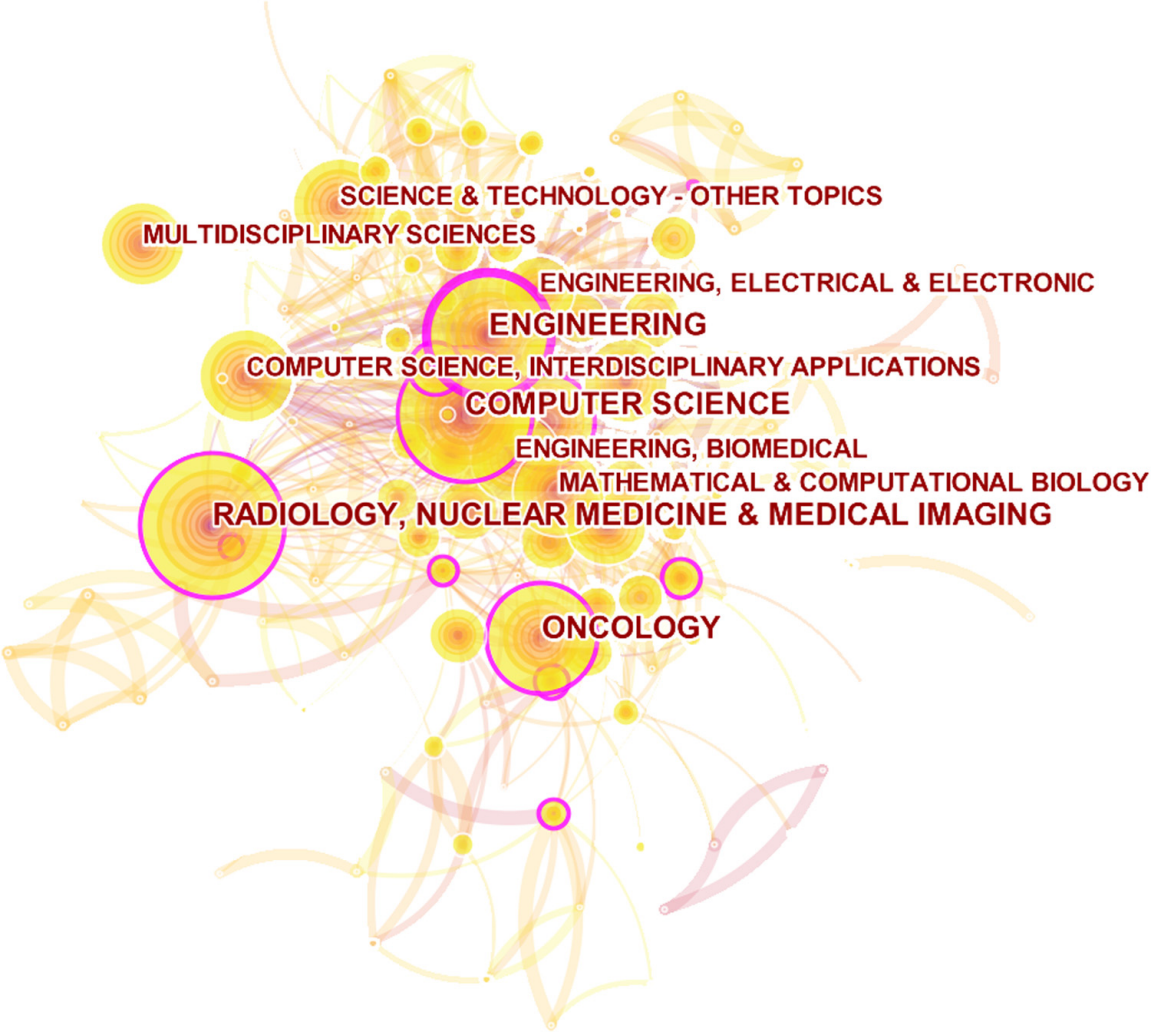
Network visualization of category co-occurrence in total literature.

Through the co-occurrence analysis of the subject in this field, network of diverse categories employed in ML and lung cancer research has been established, demonstrating the evolution of mainstream and interdisciplinary subjects in this field (Figure 5). Radiology and medical imaging is the main subject categories, because imaging is a promising tool for the diagnosis of lung cancer and has attracted attention of many scholars. Because it is an indispensable part of multi-omics analysis, this category probably tends to be more flourishing. The second is computer science, a field closely related to ML, focuses on rationale studies like algorithm design and program development. The development of oncology and engineering categories has also strengthened the application of ML in lung cancer.

### 3.5 Keyword analysis

**Figure 6:**
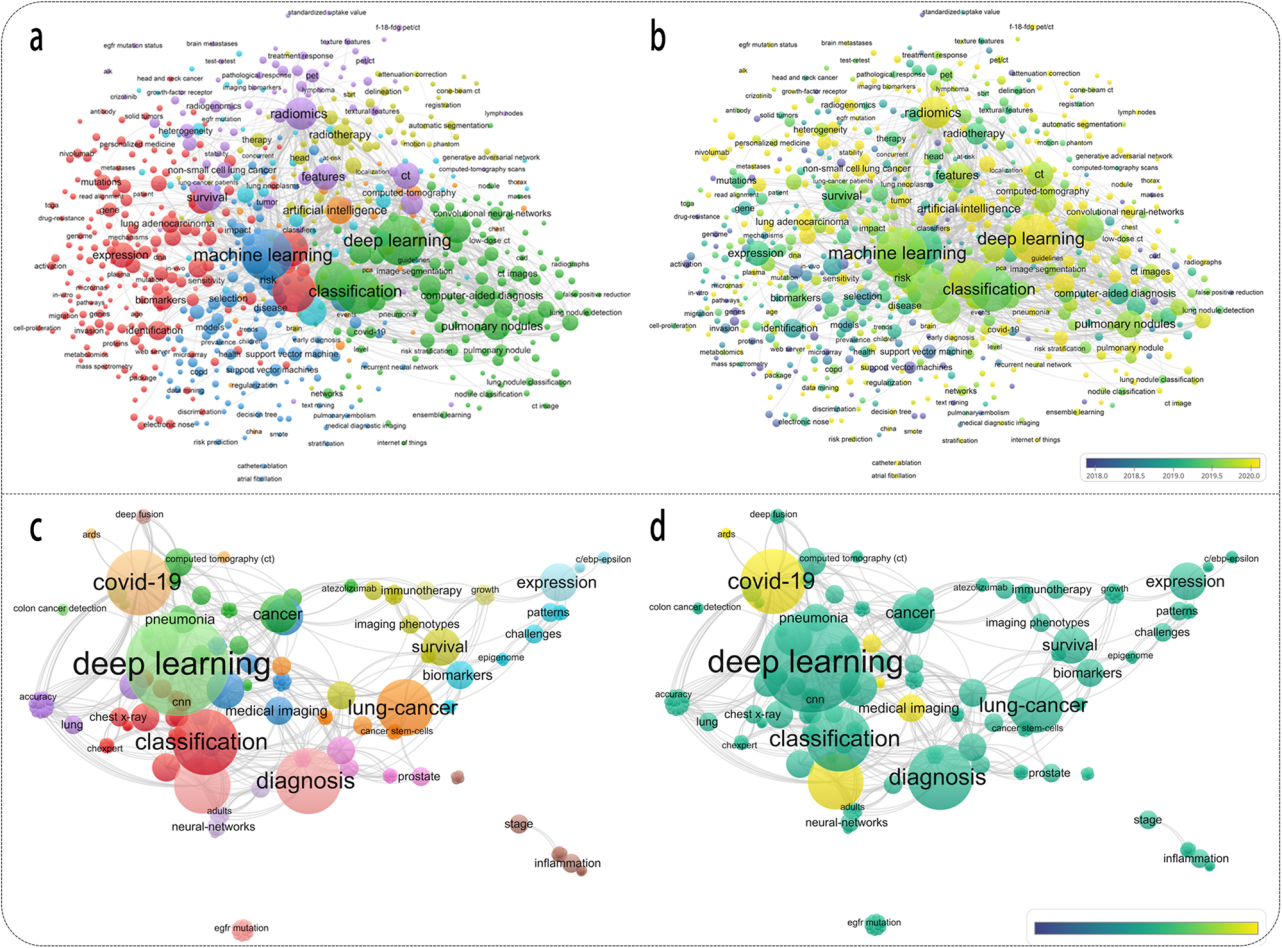
Network visualization of keyword co-occurrence in total literature and HCPs. Research field-based keyword cluster map of (a) total literature and (c) HCPs. Chronological keyword overview of (b) total literature and (d) HCPs.

Keyword is regarded as an important component of a literature and briefly summarizes its research topic. Through VOSviewer software, 679 keywords presented with frequencies of at least five were identified (Figure 6a and c). CiteSpace software were also conducted to rank the keyword according to calculated count and centrality (Table 1). Evolution in keyword as time goes by is also displayed (Figure 6b and d). During this time, the focus of research shifted from genomics to the study and application of radiomics.

**Table 1:** Top 15 keywords of ML in lung cancer

Rank	Relevant research from 2010 to 2021				Rank	HCP			
	Keyword	Count	Keyword	Centrality		Keyword	Count	Keyword	Centrality
1	Lung cancer	580	Lung cancer	0.12	1	Deep learning	58	Expression	0.24
2	ML	561	ML	0.07	2	Lung cancer	41	Deep learning	0.18
3	Deep learning	520	Classification	0.06	3	ML	37	ML	0.15
4	Classification	371	Diagnosis	0.06	4	Classification	36	Prediction	0.14
5	Cancer	309	Breast cancer	0.06	5	Convolution	31	Lung cancer	0.13
6	Convolutional neural network	208	Biomarker	0.05	6	Cancer	26	Classification	0.13
7	Pulmonary nodule	187	Radiotherapy	0.05	7	Feature	20	Cancer	0.11
8	Diagnosis	168	Support vector machine	0.05	8	Segmentation	19	Feature	0.11
9	Artificial intelligence	167	Artificial neural network	0.05	9	Diagnosis	17	Artificial intelligence	0.11
10	Model	154	Survival	0.04	10	Neutral network	17	Convolutional neural network	0.10
11	Survival	143	Prediction	0.04	11	Survival	16	Segmentation	0.09
12	Feature	142	Expression	0.04	12	Image	15	Breast cancer	0.09
13	Prediction	139	Segmentation	0.04	13	Prediction	15	Diagnosis	0.09
14	Computed tomography	139	Gene expression	0.04	14	Pulmonary nodule	14	CAD	0.07
15	Expression	137	Radiation therapy	0.04	15	Artificial intelligence	14	Neutral network	0.07

Citation burst is referred as specific literature that experienced sudden sharp rising citation time within a certain period. Emergent analysis of our topic in relevant literature was conducted through CiteSpace software (Table 2). There were 22 and 15 burst keywords identified from relevant and HCP research from 2010 to 2021.

**Table 2:** Burst keywords of ML in lung cancer

Category	Keywords	Strength	Year (time of duration)	2010–2021
A. Relevant documents from 2010 to 2021	Support vector machine	7.94	2010 (2010–2017)	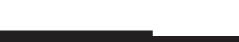
	Gene	6.47	2011 (2011–2018)	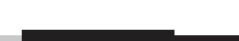
	Lung cancer	5.11	2010 (2011–2014)	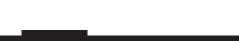
	Artificial neural network	3.87	2011 (2011–2016)	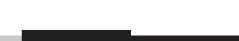
	Activation	3.15	2011 (2011–2018)	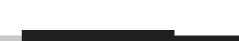
	Expression	3.6	2012 (2012–2017)	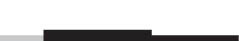
	Selection	4.28	2014 (2014–2017)	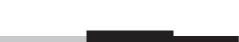
	Identification	4.72	2012 (2015–2017)	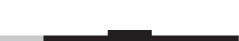
	Big data	3.67	2016 (2016–2018)	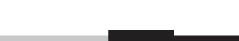
	Grade	3.19	2016 (2016–2017)	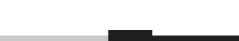
	CAD	5.2	2014 (2017–2018)	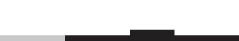
	Lung nodule	4.62	2017 (2017–2018)	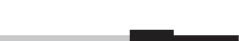
	Association	4.53	2015 (2017–2018)	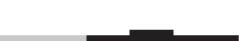
	Scan	3.61	2017 (2017–2018)	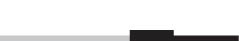
	False positive reduction	5.52	2018 (2018–2019)	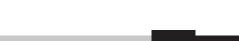
	Survival	3.74	2011 (2018–2018)	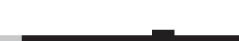
	Progression	3.61	2018 (2018–2018)	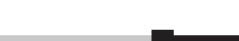
	Texture analysis	3.54	2016 (2018–2019)	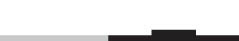
	Tumor heterogeneity	3.03	2018 (2018–2019)	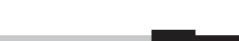
	Poor prognosis	3.01	2018 (2018–2018)	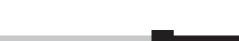
	Neural network	3.03	2019 (2019–2019)	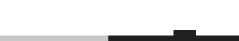
	Resection	3.2	2020 (2020–2021)	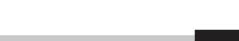
	**Keywords**	**Strength**	**Year (time of duration)**	**2012**–**2021**
B. HCPs	Expression	1.13	2012 (2012–2014)	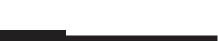
	Quantitative imaging	1.22	2015 (2015–2016)	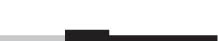
	Database consortium	1.22	2015 (2015–2016)	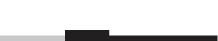
	Computational science	1.17	2015 (2015–2016)	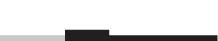
	Feature representation	1.63	2016 (2016–2017)	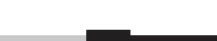
	Convolutional network	1.63	2016 (2016–2018)	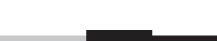
	Radiotherapy	1.27	2016 (2016–2016)	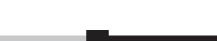
	Texture analysis	1.27	2016 (2016–2016)	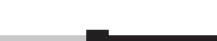
	Pulmonary nodule	1.16	2016 (2017–2018)	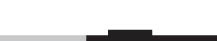
	Computer-aided detection	1.16	2017 (2017–2017)	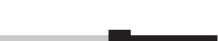
	False positive reduction	2.17	2017 (2017–2019)	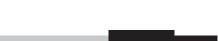
	Tumor heterogeneity	1.71	2017 (2017–2018)	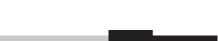
	Automatic segmentation	1.54	2017 (2017–2018)	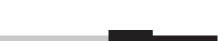
	Gene expression	1.45	2017 (2017–2018)	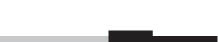
	CAD	1.45	2012 (2017–2018)	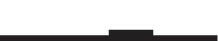
	CT scan	1.45	2017 (2017–2017)	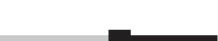
	Pulmonary tuberculosis	1.11	2018 (2018–2018)	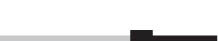
	System	1.15	2019 (2019–2019)	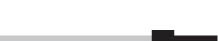
	Sensitivity	1.15	2019 (2019–2019)	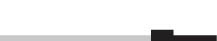
	Growth	2.13	2020 (2020–2021)	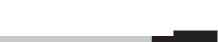
	**Keywords**	**Strength**	**Year (time of duration)**	**2017**–**2021**
C. Advances in survival prediction	Pulmonary nodule	2.02	2017 (2017–2019)	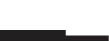
	Recurrence	1.03	2020 (2020–2020)	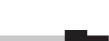
	TNM classification	0.94	2020 (2020–2020)	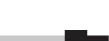
	Subtype	0.79	2020 (2020–2021)	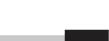
	Pattern	1.28	2021 (2021–2021)	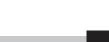

### 3.6 Literature analysis of ML-assisted survival prediction for lung cancer

Through the above analysis, we found that ML-assisted survival prediction for lung cancer might be the leading research foreland. Given that surgery is usually the treatment of choice, according to inclusion and exclusion criteria, we found that 50 articles were correlated to this refined topic for studying as well.

**Figure 7:**
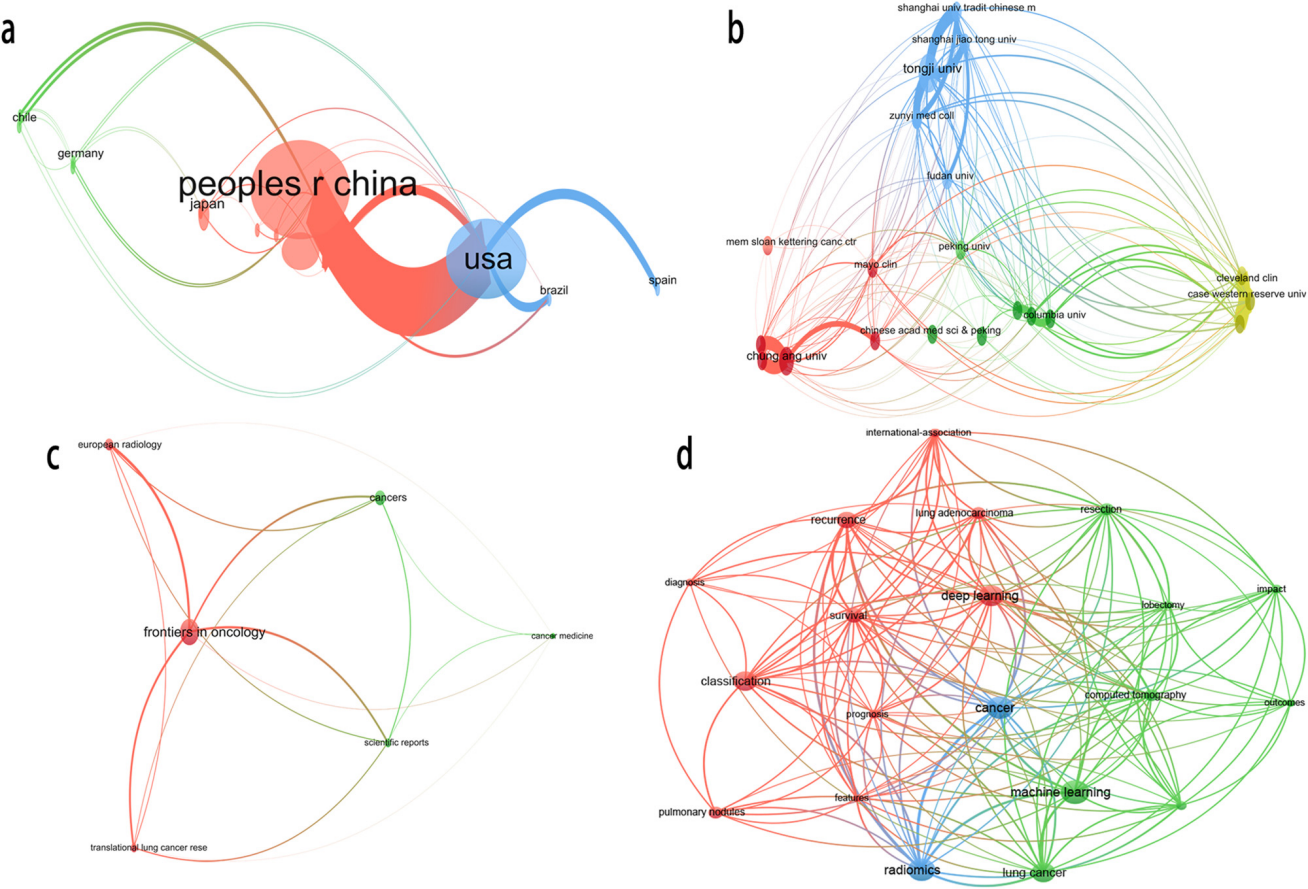
Descriptive statistical analysis of ML-assisted survival prediction topic. The cooperative network map and visualization of (a) country, (b) institution, and (c) source. (d) Keyword co-occurrence analysis of relevant literature in this field.

Publication status of this refined topic was extracted at different levels. It is shown that major literature were outputted in China (*n* = 22), and USA (*n* = 17) ranked second. Tongji university (*n* = 4) was the institution which produced most literature. The most published journal was *Frontier in oncology* (*n* = 9). The cooperative networks of countries (Figure 7a), institutions (Figure 7b), and source (Figure 7c) were visualized through VOSviewer. Even though the number of publications is relatively limited at present, it can still be seen that there was close cooperation between them.

There are 304 keywords discovered based on VOSviewer, and the co-occurrence analysis is visualized (Figure 7d). Respectively sorted by word frequency, centrality, and burstiness strength, keywords that ranked top 15 in this refined topic over the past 5 years are displayed (Table 3). The emergent analysis showed that the future hotspots were related to the differentiation and outcome of tumors.

**Table 3:** Top 15 keywords of ML-assisted survival prediction for lung cancer

Rank	Keyword	Count	Keyword	Centrality	Keyword	Burstness
1	ML	16	Lung cancer	0.27	Pulmonary	2.05
2	Lung cancer	14	Survival	0.26	Deep learning	1.94
3	Cancer	14	Feature	0.24	Pattern	1.3
4	Deep learning	14	Computed tomography	0.23	Tomography	1.26
5	Classification	13	Classification	0.21	Scan	1.18
6	Recurrence	11	Cancer	0.19	Recurrence	1.08
7	Computed tomography	10	ML	0.18	Lung neoplasm	0.95
8	Survival	9	Diagnosis	0.12	TNM classification	0.95
9	Lung adenocarcinoma	8	Lung adenocarcinoma	0.12	Subtype	0.84
10	International association	8	Pulmonary nodule	0.11	Outcm	0.76
11	Pulmonary nodule	7	Carcinoma	0.1	Classification	0.74
12	Resection	7	Outcm	0.07	Tumor	0.66
13	Feature	5	Deep learning	0.06	Shape analysis	0.66
14	Diagnosis	5	Cell lung cancer	0.06	Lung CT	0.66
15	Outcm	5	International association	0.06	Nodule characterization	0.66

## 4 Discussion

The emergence of ML has been a major impact on lung cancer research. There remain notable gaps in our knowledge of their cooperation, although several applications about healthcare have been realized [15]. Take law from Amara: “Most people are inclined to overrate a technology’s effect of in the short-term and underrate its impact in the long-term [16].” Previous relevant publication has experienced booming, and further research direction is being pioneered; quantification or modeling of massive literature to understand the overview of the research field is very necessary. To our knowledge, still no scholars have systematically sorted out the research status and evolutionary trend of our topic based on bibliometric analysis method. Herein, we for the first time performed the bibliometric of literature on ML for lung cancer published from 2010 to 2021, providing bird’s-eye view of the development trend and research frontier of this field.

### 4.1 General status of ML in lung cancer

During the established time span, an exponential growth curve starts off slowly and currently has increased faster since 2010. The fitted model indicates that the total publication will be getting higher in the next few years, so there was a growing body of active researchers already working on it. Citation-assisted background hypothesized the important literature likely to be highly cited [17]. Important literature in this field were cited more frequently so that the HCP increases year by year; finally, the overall number of citations has increased 74-fold until 2021. Surprisingly, it was found that annal citations have experienced a big boost in 2017, and saw a booming development in annual publications volume in the same, which was called golden period. According to the above assumption, this area’s importance was rising. Among all countries/regions, the USA from North America made the most output to this research domain, and its several world-famous universities like Stanford University and Harvard Medical School were also the most productive institutions. As a developing country, China and some of its institutions were not far behind in closing the gap. Both countries maintained a close academic ties; in contrast, research collaborations were relatively fragmented between other countries, which suggest that ML brought to the forefront of public attention, and its significant role in lung cancer was worth discussed worldwide. And those countries with not only progressive research institutions of medicine but also adequate medical researchers were capable to devote more resources to this topic [18]. High productivity authors tend to be the direct driving force for each field’s development [19]. A good quality literature will finally be published in appropriate journals, depending on its merit, and will be cited according to its quality [20]. Journals with high impact factor usually gain HCPs [21]. A growing number of these journals like *Medical image analysis*, *Nature communications*, and *Radiology* brought more attention to the field. It displayed the quality level of output and indicated that the radiology category has made good progress in the exploration.

### 4.2 Development trend of ML in lung cancer

Through the emergent analysis and temporal evolution of keywords, we can grant a dynamic overview of the research field. In the past few decades, with the introduction of the era of “big data”, the branches of “-omics” science mainly involved were shifted [22]. Genomics was first widely studied, gradually extended to transcriptomics, proteomics, and metabolomics in post-gene time. The public has come to realize the importance and necessity of quantitative imaging, and radiomics came into the spotlight around that time. It is widely known that there is basic prognostic information contained in pathology images. Because of substantial implications, digital pathology has become leading edge field in life science research. This reflects the development of ML’s capacity in integrating a range of “omics” data extracted from lung cancer. For clinical application, it was more focused on diagnosis, treatment, and prognosis. Applying ML to computer-aided diagnosis (CAD) of lung cancer in early stage was the focus of the research field at first, statistics revealed that gaining from advances in diagnosis approach, the 3-year survival rate for patients with lung cancer, especially non-small cell lung cancer, have exceeded 30% [1,23]. Although precision medicine (PM) heavily depends on data and analysis, ML has quickly become a key method in the development of this medical model, and it played a part for providing health care [24]. Survival prediction were a subject of great interest in recent years, as it is always the main purpose and basis of clinical decision making [25,26]. Deep learning, as a special subset of ML, has become popular in recent years, using neural network-based model to achieve powerful functionality and flexibility.

Using CAD system to interpret medical imaging statistics into another objective results, served as the “second opinion” [27–30]. Both standard eligibility criteria for screening and the mPLCOm2012 were proven to be inferior to ML model as a better tool in lung cancer detection [31]. The blossom of ML has deepened our insights into biomedicine and largely made progress in therapeutic strategy for patients with lung cancer, especially for PM [32–37]. PM referred to a healthcare model that people were considered as people at an individual level rather than a population-based approach [24]. Although classic statistical algorithms were used to assist survival prediction for clinician, like Cox proportional-hazards model, they are not precise enough [38–43]. Gains by contrast, ML has revealed its potential in predicting a patient’s survival expectancy from different omics data.

The first limitation was that use of single database and several preprocessing procedures possibly not good enough to ensure adequate and effective coverage of targeted literature. Second, many literature problems are still difficult to quantify and analysis tools and methods relatively limited, these limitations were inherent to any bibliometric approach.

## 5 Conclusion

As shown in this analysis, ML did make a big difference in lung cancer research from 2010 to 2021.
